# Supplementary material for: Simple and Rapid Assembly of TALE Modules Based on the Degeneracy of the Codons and Trimer Repeats
Source: Genes (Basel). 2021 Nov 5;12(11):1761. doi: 10.3390/genes12111761 (PMC8621181; doi:10.3390/genes12111761)
Supplement: Supplementary file 1 [file genes-12-01761-s001.zip › Supplementary files/Supplementary Figure S1.pdf]

A

CTTACACCGGATCAAGTTGTCGCTATTGCTTCTMACRDTGGTGGGAAGCAGGCATTGGAAACCGTCCAGAGACTCCTTCCCGTGCTTTGCCAAGCTCATGGA

L T P D Q V V A I A S ? ? G G K Q A L E T V Q R L L P V L C Q A H G

CTGACTCCGGACCAAGTGGTGGCTATCGCCAGCMACRDTGGCGGCAAGCAAGCGCTCGAAACGGTGCAGCGGCTGTTGCCGGTGCTGTGCCAGGACCATGGC

L T P D Q V V A I A S ? ? G G K Q A L E T V Q R L L P V L C Q A H G

CTCACCCCCGATCAGGTCGTTGCAATCGCATCCMACRDTGGCGGAAAACAAGCCCTGGAGACAGTGCAACGATTGCTGCCGGTCCTGTGTCAAGCACACGGC

L T P D Q V V A I A S ? ? G G K Q A L E T V Q R L L P V L C Q A H G

B

P1 / P3	A1	T1	C1	G1
A3	AAA	TAA	CAA	GAA
T3	AAT	TAT	CAT	GAT
C3	AAC	TAC	CAC	GAC
G3	AAG	TAG	CAG	GAG

P1 / P3	A1	T1	C1	G1
A3	ATA	TTA	CTA	GTA
T3	ATT	TTT	CTT	GTT
C3	ATC	TTC	CTC	GTC
G3	ATG	TTG	CTG	GTG

P1 / P3	A1	T1	C1	G1
A3	ACA	TCA	CCA	GCA
T3	ACT	TCT	CCT	GCT
C3	ACC	TCC	CCC	GCC
G3	ACG	TCG	CCG	GCG

P1 / P3	A1	T1	C1	G1
A3	AGA	TGA	CGA	GGA
T3	AGT	TGT	CGT	GGT
C3	AGC	TGC	CGC	GGC
G3	AGG	TGG	CGG	GGG

C

First 6 modules

Primer F	1F	2F	3F	4F	5F	6F
Primer R	1R	2R	3R	4R	5R	6R
Mixture	3×1	3×2	3×3	3×4	3×5	3×6

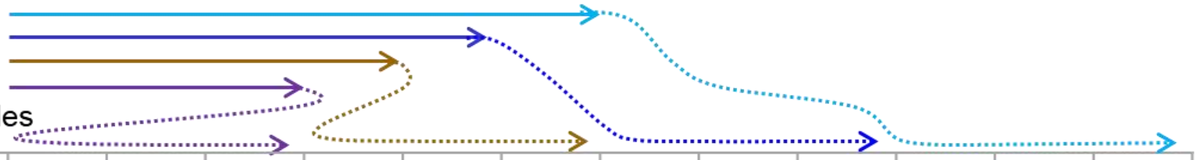

Last modules

Primer F	4F	4F	4F	5F	5F	5F	6F	6F	6F	7F	7F	7F
Primer R	R+1	R+2	R+3	R+1	R+2	R+3	R+1	R+2	R+3	R+1	R+2	R+3
Mixture	Z10	Z11	Z12	Z13	Z14	Z15	Z16	Z17	Z18	Z19	Z20	Z21

Supplementary Figure S1

X1X2, X3X4, X5X6 represent any combination of NI, NN, NG, HD respectively, there are altogether 64 kinds of combinations. MACRDT represents any combination of AACAAT, AACATT, AACGGT, CACGAT respectively, there are altogether 64 kinds of combinations.
