# Supplementary material for: Simple and Rapid Assembly of TALE Modules Based on the Degeneracy of the Codons and Trimer Repeats
Source: Genes (Basel). 2021 Nov 5;12(11):1761. doi: 10.3390/genes12111761 (PMC8621181; doi:10.3390/genes12111761)
Supplement: Supplementary file 1 [file genes-12-01761-s001.zip › Supplementary files/Supplementary Table S1.pdf]

Supplementary Table S1. Sequences of primers used for generation of trimers

Primers	Sequence
T-F :	GGTGGGAAGCAGGCATTGGAAACCGTCCAGAGACTCCTTCCCGTGCTTTGCCAAGCTCATGGACTGACTCCGGACCAAGT
T-R :	GGATGCGATTGCAACGACCTGATCGGGGGTGAGGCCATGGTCCTGGCACAGCACCGGCAACAGCCGCTGCACCGTTTCGAG
A2 :	CTGACTCCGGACCAAGTGGTGGCTATCGCCAGCAACATTGGCGGCAAGCAAGCGCTCGAAACGGTGCAGCGG
T2 :	CTGACTCCGGACCAAGTGGTGGCTATCGCCAGCAACGGTGGCGGCAAGCAAGCGCTCGAAACGGTGCAGCGG
C2 :	CTGACTCCGGACCAAGTGGTGGCTATCGCCAGCCACGATGGCGGCAAGCAAGCGCTCGAAACGGTGCAGCGG
G2 :	CTGACTCCGGACCAAGTGGTGGCTATCGCCAGCAACAATGGCGGCAAGCAAGCGCTCGAAACGGTGCAGCGG
A1 :	CTTACACCGGATCAAGTTGTCGCTATTGCTTCTAACATTGGTGGGAAGCAGGCATTGG
T1 :	CTTACACCGGATCAAGTTGTCGCTATTGCTTCTAACGGTGGTGGGAAGCAGGCATTGG
C1 :	CTTACACCGGATCAAGTTGTCGCTATTGCTTCTCACGATGGTGGGAAGCAGGCATTGG
G1 :	CTTACACCGGATCAAGTTGTCGCTATTGCTTCTAACAATGGTGGGAAGCAGGCATTGG
A3 :	GCCGTGTGCTTGACACAGGACCGGCAGCAATCGTTGCACTGTCTCCAGGGCTTGTTTTCCGCCAATGTTGGATGCGATTGCAACGAC
T3 :	GCCGTGTGCTTGACACAGGACCGGCAGCAATCGTTGCACTGTCTCCAGGGCTTGTTTTCCGCCACCGTTGGATGCGATTGCAACGAC
C3 :	GCCGTGTGCTTGACACAGGACCGGCAGCAATCGTTGCACTGTCTCCAGGGCTTGTTTTCCGCCATCGTGGGATGCGATTGCAACGAC
G3 :	GCCGTGTGCTTGACACAGGACCGGCAGCAATCGTTGCACTGTCTCCAGGGCTTGTTTTCCGCCATTGTTGGATGCGATTGCAACGAC
