# Supplementary material for: Simple and Rapid Assembly of TALE Modules Based on the Degeneracy of the Codons and Trimer Repeats
Source: Genes (Basel). 2021 Nov 5;12(11):1761. doi: 10.3390/genes12111761 (PMC8621181; doi:10.3390/genes12111761)
Supplement: Supplementary file 1 [file genes-12-01761-s001.zip › Supplementary files/Supplementary Table S2.pdf]

Supplementary Table S2. Sequences of primers used for triplet amplification

**Primers**	**Sequence**
1F	CACTGACGGGTGCCCCCCTGAACCTCACACCGGATCAAGTTGTCGCT
1R	TGAGGCCGTGTGCTTGACACAGGAC
2F	GTGTCAAGCACACGGCCTCACACCGGATCAAGTTGTCGCT
2R	GACAACTTGATCCGGTGTAAGTCCGTGTGCTTGACACAGGAC
3F	ACTTACACCGGATCAAGTTGTCGCT
3R	GGCGGGAGTCAAACCATGATCTTGACACAGGACCGGCAGC
4F	ATCATGGTTTGACTCCCGCCCAAGTTGTCGCTATTGCTTCT
4R	ATGGTCCTGGCACAACACTGGCAGCAATCGTTGCACTGTC
5F	CAGTGTTGTGCCAGGACCATGGGCTTACACCGGATCAAGTTGTCGCT
5R	ACAGAGGACGGGGAGCAGCCTTTGCACTGTCTCCAGGGCTTG
6F	AGGCTGCTCCCCGTCCTCTGTCAGGACCATGGGCTTACACCGGATCAAGTT GTCGCT
6R	GCAGCAATCGTTGCACTGTC
7F	GACAGTGCAACGATTGCTGCCTGTGCTGTGCCAAGATCATGGACTTACACC GGATCAAGTTGTCGCT
R+1	GCGAGATAACTGGGCAACAATGCTTTCCAATGCCGGCCTCCCACCA
R+2	GCGAGATAACTGGGCAACAATGCTTTCGAGCGCTGGCCTGCCGCCA
R+3	GCGAGATAACTGGGCAACAATGCTCTCCAGGGCTGGTCTTCCGCCA
